# Towards a common lexicon for equity, diversity, and inclusion work in academic medicine

**DOI:** 10.1186/s12909-022-03736-6

**Published:** 2022-10-04

**Authors:** José E. Rodríguez, Edgar Figueroa, Kendall M. Campbell, Judy C. Washington, Octavia Amaechi, Tanya Anim, Kari-Claudia Allen, Krys E. Foster, Maia Hightower, Yury Parra, Maria H. Wusu, William A. Smith, Mary Ann Villarreal, Linda H. Pololi

**Affiliations:** 1 University of Utah Health, 26 S 2000 E, 5750B EHSEB, Salt Lake City, UT 84112 USA; 2 Weill Cornell Medicine, New York, NY USA; 3 Department of Family Medicine, University of Texas Medical Branch, Galveston, TX USA; 4 Overlook Hospital at Atlantic Health, Summit, NJ USA; 5 Spartanburg Regional Medical Center, Spartanburg, SC USA; 6 Lee Memorial Health and Florida State University, Ft. Meyers, FL USA; 7 Prisma Health/University of South Carolina School of Medicine, Columbia, SC USA; 8 Sidney Kimmel Medical College at Thomas Jefferson University, Philadelphia, PA USA; 9 New York City Health and Hospitals, New York, NY USA; 10 Morehouse School of Medicine, Atlanta, GA USA; 11 Huntsman Mental Health Institute, University of Utah, Salt Lake City, UT USA; 12 The National Initiative On Gender, Culture and Leadership in Medicine: C-Change, Brandeis University, Boston, MA USA

**Keywords:** Equity, Diversity, Inclusion, Medical education, Special populations, Underserved populations, Minority faculty

## Abstract

Differential rewarding of work and experience has been a longtime feature of academic medicine, resulting in a series of academic disparities. These disparities have been collectively called a cultural or minority “tax,” and, when considered beyond academic medicine, exist across all departments, colleges, and schools of institutions of higher learning–from health sciences to disciplines located on university campuses outside of medicine and health. A shared language can provide opportunities for those who champion this work to pool resources for larger impacts across the institution. This article aims to catalog the terms used across academic medicine disciplines to establish a common language describing the inequities experienced by Black, Latinx, American Indian/Alaska Native and Native Hawaiian/Other Pacific Islander, Women, and other underrepresented people as well as queer, disabled, and other historically marginalized or excluded groups. These ideas are specific to academic medicine in the United States, although many can be used in academic medicine in other countries. The terms were selected by a team of experts in equity, diversity, and inclusion, (EDI) who are considered national thought leaders in EDI and collectively have over 100 years of scholarship and experience in this area.

## Background

Minority or (minoritized) faculty experiences are well documented in the academic medicine space as well as on the greater health sciences campuses [1–6]. These experiences have been collectively called “taxes,” or “taxation” and they are disproportionately felt by underrepresented faculty in academic medicine regardless of institution type [1, 6]. Examples of these experiences include: minoritized faculty participating in more unpaid diversity efforts, being the targets of racism, isolation, lack of honest effective mentorship, increased clinical responsibilities when compared to non-minority peers, and being considered for promotion later and less often than their non-minoritized peers. These taxes are also described as a subsidy for non-underrepresented faculty in academic medicine or a “majority subsidy” [1]. With increasing efforts at universities across the country to address issues of equity, diversity, inclusion and anti-racism, it has become clear that there is a need to establish a common vocabulary and shared understanding for faculty and other individuals conducting research in this area. Much of the literature, as well as these terms, is grounded in the context of academic medicine in the United States, and the authors recognize that all terms will not have the same use in all academic medical settings. While minoritized medical students surely experience some of the same inequities, we will limit this paper to the faculty experience.

## Construction and content

This article aspires to introduce a basic glossary to present terms common to all areas of the academic medical environment and ensure a common meaning. Our glossary defines frequently referenced terms and concepts in equity, diversity and inclusion work that are used in a variety of academic and non-academic settings. The terms were selected by a team of experts in equity, diversity, and inclusion, (EDI) who are considered national thought leaders in EDI and collectively have over 100 years of scholarship and experience in this area. In addition, we have included a compilation of terms that have been introduced into the literature by the authors. (KMC, JCW, LHP, JER, WAS). When viewed as a whole, these terms will help equity, diversity, and inclusion scholars and officers alike find a common lexicon to describe the minority faculty experience and to ensure that equity outcomes are achieved in academic medical and health sciences settings.

## Glossary of Concepts in Equity, Diversity and Inclusion in Academic Medicine

### Academic redlining in medicine

The term “redlining” is associated with the historical practice of outlining areas with sizable Black populations in red ink on maps where banks and the Federal Housing Administration (FHA) denied mortgage and insurance products [2]. “Redlining” now extends to discriminatory practices of denying or charging more for a broad range of services, including banking, insurance, health care, consumer products, and education to marginalized communities. In health care, an example of a redlining practice is the inequitable geographic distribution of health services and facilities within marginalized communities [3]. This disparity results in decrease access, poorer health outcomes, and increased out-of-pocket costs such as transportation, time missed from work, and childcare expense to seek healthcare. The decreased concentration of health services and facilities creates health deserts like food deserts in areas that have less access to grocery stores, thereby limiting access to affordable nutritious food. Academic redlining refers to the systematic exclusion of students from underrepresented backgrounds from entry into medicine using standardized test hard cutoffs, such as the Medical College Admissions Test (MCAT). The use of an arbitrary cutoff contributes to the persistent lack of diversity in medicine [4].

### Bias

In academia, biases are preconceived notions about individuals or groups that could be based on stereotypes, racism, sexism, or other forms of oppression. Biases allow their users to take “cognitive shortcuts” instead of learning about the individuals or groups. Individual biases are snap judgements that can lead to inappropriate decisions and discriminatory, oppressive practices [5]. Unconscious or implicit biases are a sub-set of bias and will be addressed here.

### Unconscious or implicit bias

Unconscious bias, also known as implicit bias, refers to attitudes or stereotypes that are outside our awareness but affect our understanding, our interactions, and our decisions [6]. Much attention has been given to unconscious biases, namely because they lead to, in effect, racist, sexist or other oppressive actions by offenders who do not identify as racist, sexist, etc. Regardless of intention, unconscious biases hurt people of color, and universities across the world are working on the elimination of this form of bias.

### Deficiency model

The framework stemming from ingrained racist ideologies that suggest Underrepresented Minority (URM) faculty professional development is needed due to deficiencies in the faculty member rather than the deficiencies of the institution in regard to inclusivity, racist policies, and equity [7, 8]. It includes the bias that predominantly white institutions (PWI) are devoid of institutionalized racism due to the longstanding history of white privilege as the norm [9, 10]. A similar term, *deficit model*, refers to the skills that some faculty may lack due to multiple factors [11–13], but the deficiency model terminology is person based and not skills based. This comes from the historical concept that minority faculty are deficient, essentially an extension of racist ideas that have permeated academia for millennia [14, 15].

### Disparity

Disparity means difference. In healthcare and in academia more broadly, the term disparities are usually used to describe differences in outcomes between groups. For example, a promotion disparity would be described when comparing women and men in the same discipline and noticing that women are promoted less often [16]. A healthcare disparity would be one where women of color die at higher rates of breast cancer when compared to white women [17, 18]. Disparities are a hallmark of our society, and elimination of disparities moves us towards a more equitable society.

### Distance travelled

Distance traveled describes differences among faculty in the path to their present position; for many URM faculty, arriving at this position entailed more tasks than for non-URM faculty [19]. This may mean having to work during high school, college, or even medical school to help meet financial obligations at home; taking extended leaves of absence to care for loved ones; dealing with health, legal, or educational challenges that impede academic progress, etc. These challenges may extend time to degree beyond four years for undergraduate and/or medical school or beyond eight years for both. Faculty who have overcome these additional challenges are said to have a greater distance travelled when compared to those who did not experience these challenges.

### Gate blocking

Gate Blocking describes the result of institutional actions and institutional neglect in the setting of institutional racism that essentially blocks the gate of progress or advancement for underrepresented faculty in *academic medicine *[19]. Underrepresented minority faculty are gate blocked from promotion and tenure and leadership opportunities and can be subjected to pseudo-leadership and minority and gratitude taxation (see below). Institutional action or inaction leads to feelings of imposter syndrome and reverse imposter syndrome for underrepresented minority faculty [19]. This phenomenon can be experienced in any situation where academic promotion is controlled by institutional leaders, and underrepresented minority faculty can be tokenized. Not only does this group suffer from lack of advancement as a result of being gate blocked, they may also leave academic medicine [19].

### Imposter syndrome

Those who have imposter syndrome doubt their abilities or accomplishments, and fear being exposed as a fraud because of consistent messages that they don’t deserve success because of one or all their identities. This happens even in the situation where they are the most qualified for the position they occupy [20]. First coined as a phenomenon observed in white women serving in higher education [21], This phenomenon is also observed in underrepresented minority faculty in academic medicine and across higher education, regardless of gender identification [19, 22]. Imposter syndrome steals energy from achievement and consumes it in self-doubt, and other damaging pursuits. The mental health implications can shorten careers, frustrate individual faculty members, and keep faculty members from aspiring to higher positions [23].

### Microaggressions

This social phenomenon was first described by Chester Pierce, M.D., a prominent Harvard-trained Black psychiatrist: “The subtle, cumulative mini-assault is the substance of today’s racism” [24]. Microaggressions are the regular or daily experiences that carry messages of insult due to group membership. These are everyday slights, indignities, and put-downs that members of marginalized groups experience in their everyday interactions. Individuals who perpetrate the microaggressions are often unaware that they have engaged in an offensive or demeaning way. Wing Sue has significantly expanded this work to address various forms of microaggressions, suggests a process model, and discusses engagement in the workplace or classroom. Wing Sue’s work has shown that microaggressions are not limited to race/ethnicity or faculty situations, and the expansion of this definition to women, lesbian, gay, bisexual, transgender, queer (LGBTQ +) individuals, and others serves to increase awareness and understanding of the damaging effects for multiple marginalized groups [25–27].

### Motherhood penalty

Women who become mothers sacrifice career progress, lose wages, and are stigmatized, while men are rewarded [28]. Early experimental work by multiple scholars showed that in addition to the above challenges, mothers also are perceived as less committed to their jobs [29–31].

### Power distance

Underrepresented faculty in *academic medicine *are more likely to be in entry-level rank and less likely to be tenured. Power distance refers to the distance between a traditional junior faculty member or other entry-level position and senior leadership. This distance may cause the underrepresented faculty member to defer to the opinions, ideas and plans of senior leadership even when those plans may be at odds with their own beliefs and potentially harmful to their career” [22].

### Professional gaslighting

Gaslighting is a psychological manipulation that causes an individual or group to question their own sanity or perception of reality. First used in a play and a film called “Gas Light” [32], the term was applied to sociology in the 1960s as pertaining to intimate partnerships, the term has evolved to describe any toxic dynamic in which an individual/group with power and control consciously or unconsciously deceives a targeted individual/group, causing them to question their own judgement. This can lead to burnout, insecurity, and an inability to maintain a stable and thriving career [33]. In *academic medicine*, gaslighting creates cognitive dissonance or low self-esteem in the underrepresented minority faculty member, and can exacerbate imposter syndrome, isolation, and emotional destabilization. Gaslighting often manifests in the form of denial, misdirection, and misinformation. It gradually erodes the faculty member’s confidence, causing them to question their role in and contributions to the department.

### Psychological vulnerability

Psychological vulnerability causes URM and women faculty to limit their contributions and constantly edit their thoughts, their words, and their actions. This happens in the absence of psychological safety, the shared belief held by members of a team that the team is safe for interpersonal risk taking [34]. An example would be URM faculty having a meeting outside of the department, or even outside of the institution to avoid psychological vulnerability.

### Racial battle fatigue

First coined in 2003 by Dr. William A Smith at the University of Utah, this describes the results of natural race-related stress responses to distressing mental and emotional conditions. Racial battle fatigue is a systemic race-related repetitive stress injury. Consequently, poor health or illness can emerge from constantly combating biopsychosocial factors experienced as racially discriminatory, dismissive, demeaning, insensitive, hostile, or violent. This injury was first described as a phenomenon that affected Black faculty on predominantly white campuses but has evolved to describe the experiences of all racially marginalized and underrepresented people, irrespective of their interlocking identities. According to Dr. Smith, “racial battle fatigue helps to explain the causes, manifestation, and pre-mature deaths of targets of racism” [35].

### Racism

A system of discrimination against Black and other people of color based on perpetrators ‘ perception of the victim’s phenotype. In the United States and worldwide, the direction of racism as a system is anti-Black, or anti-Asian, etc. Racism manifests itself in many ways, but always advantages one race over another [36, 37].

### Institutionalized or structural racism

Sometimes called “racism without racists” it is the racism that is perpetuated by policies and institutional governance systems that favor white men and women over all others [37]. The evidence for this is in the low representation of women of color in leadership, the small percentage of Asians in medical institution leadership when they represent 25% of all medical faculty, and the small percentage of URM faculty in academic settings across the United States. Institutionalized racism is also manifest outside of the academic setting. Historical examples of redlining and Jim Crow laws are examples of institutional racism at its strongest. Yet, even today, it is manifest in higher mortgage rates for Black and Latinx borrowers, efforts across the U.S. to silence minority voters through stricter voter registration laws, and the association between low socioeconomic status and race. While these systems of oppression were not invented by anyone alive today, they are still perpetuated through institutions. Institutionalized racism also has a role in health disparities, as medical professionals throughout the world are taught that Black race is associated with higher risk for certain diseases and is associated with poorer health outcomes.

### Reverse imposter syndrome

Describes how underrepresented minority faculty can feel when they are made pseudo-leaders at the hands of institutional racism, by academic institutions. In contrast to imposter syndrome, in reverse imposter syndrome underrepresented faculty are tokenized and placed in leadership roles only for the diversity they bring when they are not trained, prepared, or supported for such an opportunity. This tokenization opens them up to manipulation by senior leaders due to limited knowledge and training for the leadership role. As reverse imposters, underrepresented minority faculty may forego trainings and opportunities that would promote their growth in skills within their current institution.

### Stereotype threat

A psychological phenomenon where an individual’s performance in a task is affected, often adversely, due to the fear or anxiety of confirming a negative stereotype about how that group will stereotypically perform in that task [38]. Rooted in the work of Claude Steele and Joshua Aaronson, numerous psychology experiments have documented examples:

- When primed with the stereotype of underperformance, People of Color and Women underperformed on standardized tests compared to White People and Men.
- White people underperformed in athletic tasks compared to Black people.
- White people underperformed in cognitive tasks when compared to Asian groups, especially in STEM subjects.

Steele’s most recent book, *Whistling Vivaldi*, summarizes many of these experiments and addresses strategies to address stereotype threat, including a) “priming” those at risk of stereotype threat with information about the threat and expressing confidence in their ability to overcome it, and b) having counter messages in the environment that suggest a sense of welcome [39–41].

### Tokenism

The practice of making only a perfunctory or symbolic effort to do a particular thing, such as by recruiting a person from an underrepresented group only to prevent criticism and give the appearance that people are being included or treated fairly [42, 43]. Tokenism is designed to show that an organization values equity, diversity, and inclusion. Frequently the most junior or the most professionally vulnerable URM faculty member is invited to participate in leadership discussions, with leaders understanding that junior voice from a URM background is easily manipulated or controlled [44]. Tokenism is also used as an excuse not to include URM voices in leadership because leaders do not want to “tokenize.” The antidote for tokenization is to have multiple URM voices at the highest leadership levels and on decision making boards.

### White manning

White manning is behavior that some white men exhibit that ignores both humility and the vulnerable and allows him to pretend that he is the sole expert on all things. This attitude led to immediate closure of college campuses at the start of the COVID19 pandemic without considering the needs of minoritized and marginalized students. In *academic medicine*, it allowed for a delayed response of many hospital systems across the US including the failure to see the vulnerability of many frontline workers who were predominantly African American and Latinx. “White manning” justifies the continued lack of diversity in the physician work force. It also blames URM students, residents and faculty for their lack of representation and not systemic racism [45].

### Subsidies for non-minoritized faculty

#### Citizenship tax for women

Uncompensated work-related duties that require dedicated time often performed at work but often on off hours. These duties are less likely to contribute to career advancement. Some examples are posing for brochure pictures, taking notes at meetings, participating in, and organizing social events, committee participation etc. Women are asked to do more citizenship tasks than men and feel that gender plays a significant role [46, 47].

#### Cultural taxation

Originally coined by Dr. Amado Padilla, this term refers to the additional, uncompensated burdens placed on minority faculty in academic settings [48]. Some of these additional burdens have to do with uncompensated diversity work, pressures to be the spokesperson for the institution without compensation, etc. This “taxation “ is the model for the other “taxes “ listed in this paper and has been used to describe additional burdens for women, for racial/ethnic minorities, etc.

#### Gratitude tax

This tax refers to the learned attitudes of URM faculty members from lived experience that they should be “grateful” that academia has “given them a chance” or “taken a risk” in hiring them [49]. It keeps URM faculty from asking for resources and time that are allotted to non-URM faculty because, if you do ask, you are not grateful for your chance. This tax can result in additional service work or responsibility taken on to show thanks or commitment to another person or a team. It can also make it difficult to say “no” as there is the potential for being perceived as “ungrateful” if that extra work is rejected, no matter the reason [49]. This work is taken on even if the individual is no longer experiencing professional growth. As a result, the individual may avoid seeking new opportunities and delay academic advancement.

#### Invisibility tax for women

Most pronounced for Black women, this refers to the exclusion of the experiences of women of color from discussions of women in academia. Woman in academic circles usually means white woman [50]. In addition, white women also are made to feel invisible and unvalued in many academic spaces, particularly in male-dominated disciplines [51].

#### Minority tax

The additional burden of responsibility and expectations placed on underrepresented minorities than those who identify with the dominant culture; all in addition to coping with and managing daily, institutionalized bigotry in professional and personal lives. “The proportion of Black, Latino, and Native American faculty in US academic medical centers has remained almost unchanged over the last 20 years. This tax is, in reality, very complex, and a major source of inequity in academic medicine. The “minority tax” is better described as an Underrepresented Minority in Medicine (URMM) faculty responsibility disparity. This disparity is evident in many areas: diversity efforts, racism, isolation, mentorship, clinical responsibilities, and promotion” [52].

#### Minority woman tax

The intersection of woman taxes and minority taxes. For most, this is an exponential increase, and not a simple summation. These include tokenism, gratitude taxation, and the vulnerabilities that are magnified by sexism and racism [50, 53]. This minority woman tax has also been described by Hishfield and Joseph as identity taxation for women, especially women of color in academia [54].

#### Women’s pay disparity

Women with the same experience, productivity, and clinical expertise are paid less than men. In the United States, women physicians earn 75 cents on the dollar compared with their male counterparts, even after accounting for numerous potential confounders. The 2020 numbers show the same disparity despite the increase in overall compensation. Many women, because of competing demands, are being paid for 80% time but continue to perform at 100% time. Men who work part-time usually do so to fulfill another career goal while women do so to care for children or elderly parents. At many institutions, reduction in time commitment leads to a disproportionate decrease in benefits like healthcare coverage and lack of opportunity to move into leadership positions. This also diminishes the productivity in scholarship due to being assigned uncompensated service-related tasks [55]. There is also a documented gender gap in National Institutes of Health (NIH) grant applications and funding [56–58].

## Utility and discussion

This glossary was designed to create a common vocabulary for those interested in equity, diversity and inclusion work in academic medicine and the health sciences, with a focus on the work done in the United States. While this is not designed to be an exhaustive list, it has value as it combines terms that are frequently used, but not always understood in the same way. This glossary can be used in training and onboarding for academic leaders to ensure that experiences common to minoritized faculty are named and can open a pathway for deeper understanding of faculty who have been “othered.” In the United States, amid increasing divides in political and cultural thought, a common vocabulary can serve as a nidus for unity. The terms and the examples provided can also help leaders recognize behaviors and attitudes that are detrimental to the academy and find ways to eliminate them. In addition, the taxation and the other forms of oppression listed could serve as an impetus for policy and institutional change to ensure that our institutions of higher learning become bastions and leaders in equity. Institutional commitment to the elimination of the inequities described can be facilitated by dialogue, but true change happens when the policies that govern behavior are changed to address the phenomena described in this lexicon.

## Conclusions

Equity, Diversity, and Inclusion (EDI) work in academic medicine has been studied for decades, and due to the pandemic and the subsequent aftermath of racial justice protests in the United States and internationally, it is receiving renewed attention. Because it is a rapidly changing field, this update can serve academic medicine leaders as they seek definitions and a common vocabulary to continue this work. Mitigation strategies and behaviors that individuals and institutions can use to address the phenomena defined by the terms in this work are provided in Table 1.

**Table 1:** Mitigation strategies for selected observed phenomena

Observed Phenomena	Mitigation strategies for Individuals and Institutions
Academic Redlining	Serve on admissions committees. Identify and advocate for minoritized students that may otherwise be excluded by automatic cut offs Adopt more holistic admissions criteria, including recognition of distance travelled by students, and examine applications from all minoritized students in your applicant pool. Implement bias training for all admissions committee members at regular intervals (every 2–3 years)
Deficiency Model	Refer to all academic disparities between minoritized and non-minoritized groups as educational system failures and not individual failures Provide opportunities for minoritized students that address system failures (tutoring, test preparation, etc.) and ensure that those opportunities are available to all students
Gate Blocking	Support and encourage minoritized junior faculty to rise through the ranks with opportunities to gain the necessary skills to ensure any administrative work, (especially equity, diversity, and inclusion work), is counted for their career advancement and is in harmony with their career goals. Support should include professional coaching, faculty development, direct mentorship, and a commitment of time and financial resources toward their professional work
Invisibility tax	Establish a women’s advisory council with direct responsibility to and authority from the president of the university Create awards, leadership positions, and events that honor, promote, and recognize the invaluable contributions of individual women faculty, and women in medicine as a group
Citizenship tax	Ensure that citizenship tasks are equitably distributed among the faculty. Many of these tasks may better serve the institution in the professional staff space. Specifically examine the percentage of citizenship tasks that are performed by women and redistribute as needed
Gratitude Tax	Review committee composition to ensure URM faculty are included and equitably represented and can share dissenting opinions in a psychological safe space Recognize when URM faculty agree with Academic Health System leaders out of convenience or fear and create opportunity to determine if true agreement exists or if this group is agreeing because of institutional or organizational hierarchy or the gratitude tax [19, 49, 52].
Professional Gaslighting	When a URM faculty member expresses feelings of burnout, micro-aggressions, or disorientation, believe them and use departmental resources to support them and correct the underlying cause of their misaligned experience. Consider monitoring URM faculty members for signs of isolation or misalignment and engage with them through mentorship and sponsorship that is sensitive to their unique needs in the academic space

We encourage faculty, staff, administrative and academic leaders to study the terms, and to implement mitigating strategies based on what they learn and see at their individual institutions. Together, we can become the change we seek.

## Data Availability

The data that was used to create this manuscript is readily available through existing databases, including PubMed and ERIC.

## Abbreviations

EDI: Equity, Diversity, and Inclusion
FHA: Federal Housing Authority
MCAT: Medical College Admissions Test
URM: Underrepresented Minority
PWI: Predominantly White Institution
LGBTQ +: Lesbian, Gay, Bisexual, Transgender, Queer
NIH: National Institutes of Health
URMM: Underrepresented Minority in Medicine
